# Mesoscale Simulations of Structure Formation in Polyacrylonitrile Nascent Fibers Induced by Binary Solvent Mixture

**DOI:** 10.3390/ijms24119312

**Published:** 2023-05-26

**Authors:** Pavel Komarov, Maxim Malyshev, Pavel Baburkin, Daria Guseva

**Affiliations:** 1 Scientific Research Department, Tver State University, Zhelyabova 33, 170100 Tver, Russiaguppi_oc@mail.ru (P.B.); 2 A.N. Nesmeyanov Institute of Organoelement Compounds RAS, Vavilova Street 28, 119991 Moscow, Russia; guseva@polly.phys.msu.ru

**Keywords:** mesoscale simulations, coarse-grained model, dynamic density functional theory, polyacrylonitrile, high-tech textile, coagulation, multiphase polymer

## Abstract

Polyacrylonitrile (PAN) is widely used as a raw material for the production of high-modulus carbon fibers, the internal structure of which is directly affected by the spinning of the precursor. Although PAN fibers have been studied for a long time, the formation of their internal structure has not been sufficiently investigated theoretically. This is due to the large number of stages in the process and the parameters controlling them. In this study, we present a mesoscale model describing the evolution of nascent PAN fibers during the coagulation. It is constructed within the framework of a mesoscale dynamic density functional theory. We use the model to study the influence of a combined solvent of dimethyl sulfoxide (DMSO, a good solvent) and water (a non-solvent) on the microstructure of the fibers. A porous structure of PAN is formed as a result of the microphase separation of the polymer and the residual combined solvent at a high water content in the system. The model shows that one of the possible ways to obtain the homogeneous fiber structure is to slow down the coagulation by increasing the amount of good solvent in the system. This result is in agreement with the existing experimental data and confirms the efficiency of the presented model.

## 1. Introduction

Polyacrylonitrile (PAN) has a low density, thermal stability, high strength, elastic modulus, and chemical stability. After functionalization with different chemical groups, it can form stable materials with different structures. These properties have established PAN as essential material in chemical and textile industry, for foods packing materials, electronics, and cosmetics [1,2]. PAN fibers are also used for outdoor awnings and clothing, and they play a key role in high-tech applications such as air/water filters [3,4]. They are also utilized as a precursor for the manufacturing of carbon fibers (CFs) due to its good mechanical properties, high carbon content, and ability to undergo cyclization reactions at high temperatures [2,5,6,7,8,9,10,11,12,13,14]. CFs are widely used in high-tech applications where heat resistance, chemical inertness, and high tensile strength are required [5,6,7,8,15,16,17]. Their formation is accompanied by complex physical and chemical transformations in the precursor, triggered by the influence of solvent quality, mechanical stress, and temperature. The supramolecular structures (nanofibrils, networks, crystals, etc.) formed during the multiphase transition have a decisive influence on the properties of the final product. It is believed that the properties of CFs can be controlled by varying the formation conditions of precursor fibers [6,9,14,16].

One of the most effective ways to form the desired structure of a polymeric material is to perform a controlled phase transformation in polymer solutions [18,19,20]. Wet and dry–wet spinning processes for the production of PAN fibers are well suited for this purpose [5,7,12,15,16,21]. In these technologies, the spinning solution (polymer solution in a good solvent, e.g., dimethyl sulfoxide) is extruded through a spinneret into a bath containing a non-solvent (i.e., poor solvent) for the polymer. Thus, the primary structure of the PAN fibers is formed in the course of the diffusive exchange between the spinning solution jet and the coagulation bath [22]. Freshly coagulated fibers typically have an unoriented fibrillar network structure with numerous pores, capillaries, and voids [9,23,24,25], which can be arranged radially in the fiber cross-section. The capillaries and voids can further provoke the formation of microcavities and inhomogeneities of the CFs, which prevent the achievement of high mechanical properties. In this way, the characteristics of the coagulation process determine the quality of the microstructure of both the as-spun fiber and the resulting CFs. Thus, the main difficulty is to choose the optimal values of the parameters to obtain an optimal PAN fiber structure.

The diffusion exchange taking place in the coagulation bath had already attracted the attention of many researchers [22,26,27,28,29,30]. Although the general features of this process are well known, the application of experimental studies and theoretical methods to accurately observe and describe the structural transformations that occur in nascent chemical fibers is very challenging. In the case of using theoretical methods, the main difficulty is associated with a variety of parameters, which should be taken into account simultaneously. Thus, the structural transformations in the nascent fibers are not fully explored.

Most models describing the fiber formation process are based on the solution of the diffusion equations [26,27,30,31]. Their reasonable use allows us to learn the main aspects of the kinetics of mass exchange between the fiber and the coagulation bath. At the same time, their main parameters have to be adjusted using experimental data. To the best of our knowledge, there is currently no fully experimental or theoretical method for obtaining details of the structure formation in the nascent fiber at a molecular level. In this case, computational modeling based on the use of atomistic and mesoscale methods is a convenient tool to reveal the organization of materials at the highest level of resolution and to directly observe the trends of the occurring processes. A good overview of these methods can be found in [32]. Designing well-functioning models gives us a tool to predict the optimal conditions for obtaining the desired properties of the fiber, thus reducing experimental costs. Therefore, the development of computational models of different stages of chemical fiber formation is a relevant objective.

The literature search also shows that a direct computational study at the atomistic and coarse-grained levels has never been used to describe the process of PAN coagulation. Articles presenting results on PAN modeling include studies of the interaction of polyacrylonitrile with solvents [33], the behavior of a single PAN chain in solution [34], and the interaction of PAN chains with carbon nanotubes in solution [35]. We can also mention the work on PAN carbonization [36,37,38,39,40]. In this light, our research fills this gap in the simulation of chemical fibers.

Based on our previous experience in studying structure formation in various molecular systems, we chose the mesoscale modeling methodology as a working tool [41,42,43]. A specific application of the developed model is aimed at studying the influence of the composition of the system, namely, the ratio of polymer to solvent, and the effect of the quality of the solvent on the structure of PAN nascent fibers. To build and implement the model, we use Dynamic Density Functional Theory (DDFT) [41,44], which we have previously applied to study other systems [45,46,47]. DDFT is a highly efficient mesoscale method for soft matter modeling. It allows us to study melts and solutions of macromolecules on relatively large scales of ~200 nm and time intervals of ~1 μs, which are difficult to achieve at the atomistic level of simulations. DDFT also allows us to simulate shear flow, which is useful for studying the formation of a fiber structure under tension.

The rest of the article is organized as follows. The results obtained are discussed in Section 2. The limits of our model and the portability of the results to other systems are discussed in the Section 3. Section 4 gives brief information on the modeling method used and a description of the model construction, its parameterization and provides information on the methodology of simulations. Finally, we summarize the results of our work in the Section 5. Additional information can be found in the Supplementary Materials file.

## 2. Results

**Figure 1:**
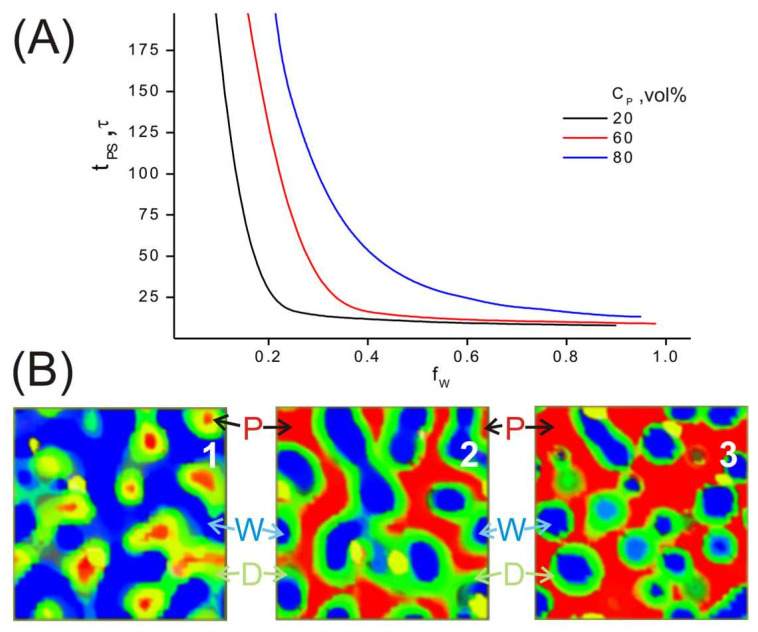
Effect of PAN content on microphase separation time and system structure in the final state (*t* = 200 Δ*t*–5000 Δ*t*). (**A**) Microphase separation time as a function of water content in the combined solvent composition. (**B**) Distribution of PAN, DMSO, and water densities in the simulation cell: (1) *C*_P_ = 20 vol%, *f*_w_ = 0.77; (2) *C*_P_ = 60 vol%; *f*_w_ = 0.77; (3) *C*_P_ = 80 vol%, *f*_w_ = 0.7. Red color corresponds to the PAN matrix (ρ_P_ > 0.8), green color corresponds to DMSO (ρ_D_ > 0.02), and blue color corresponds to water (ρ_W_ > 0.8). The following abbreviations are used here and in the Figures below: P-PAN, D-DMSO, W-water.

First, we performed calculations for systems with different PAN volume fractions *C*_P_ of 20 vol%, 60 vol%, and 80 vol% and different water contents in the composition of the combined solvent *f*_w_ = 0–1 (*f*_w_ = *C*_W_/(*C*_D_ + *C*_W_), where *C*_D_ and *C*_W_ are DMSO and water volume fractions). Note that the following abbreviations are used in the text and Figures: P-PAN, D-dimethyl sulfoxide (DMSO), W-water. The rate of microphase separation increases with increasing water content in the combined solvent (see Figure 1A). Due to the large difference in the values of the solubility parameters δ_P_ and δ_W_ at a low percentage of PAN (*C*_P_ = 20 vol%), the polymer forms isolated domains in the final state (see Figure 1B, snapshot 1). As C_P_ increases, the observed picture is reversed. In this case, water begins to form isolated domains embedded in the polymer matrix. At their boundary, a transition layer of DMSO is formed (see Figure 1B, snapshot 3). At the same time, DMSO (in all cases) remains in the domains formed by the polymer, because its solubility parameter is close to that of PAN.

**Figure 2:**
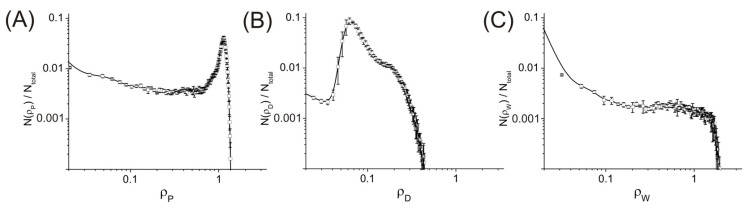
Fraction of system regions with density ρ for (**A**) PAN, (**B**) DMSO, and (**C**) water. *N*(ρ) is the number of nodes of the grid where the density is equal to ρ, and *N*_total_ is the total number of nodes of the grid in the simulation cell. *C*_P_ = 80 vol%, *f*_w_ = 0.5.

Figure 2 shows an instance of the distribution profiles *N*(ρ_α_)/*N*_total_ characterizing the volume fraction of the simulation cell regions where the density of the components of the system (P, D, and W) is equal to certain values of densities fields ρ_α_ (α = P, D, W) for the system with *C*_P_ = 80 vol%, *f*_w_ = 0.5. See Section 4 for a description of ρ_α_. As can be seen, the distribution *N*(ρ_α_,*t*)/*N*_total_ for PAN has a clear maximum at ρ_P_ ≈ 1.02. The figure also shows that DMSO does not form regions of high density.

**Figure 3:**
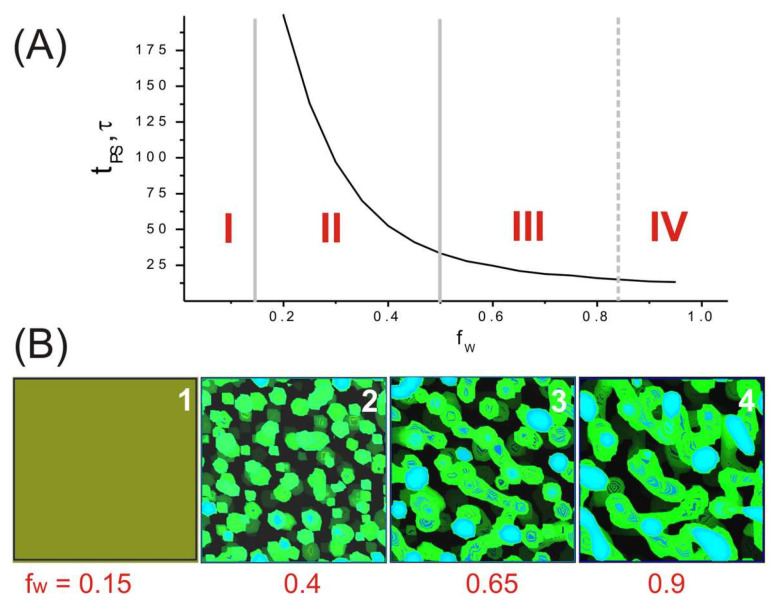
(**A**) Dependence of the microphase separation time on the water content at a fixed PAN volume fraction. (**B**) Visualization of the voids in the PAN matrix in the final states and different water contents (ρ_W_ > 0.79, green and blue colors). *C*_P_ = 80 vol%, *f*_w_ = 0–1. In (**A**), region: I corresponds to PAN + DMSO homogeneous mixture (*f*_w_ < 0.16), snapshot 1 in (**B**); II—small spherical domains formed by water (0.16 < *f*_w_ < 0.5), snapshot 2; III—dumbbell domains (0.5 < *f*_w_ < 0.85), snapshot 3; IV—water domains forming percolation network (0.85 < *f*_w_), snapshot 4.

Figure 3A shows that the DMSO:water ratio has a strong influence on the rate (*t*_ps_ decrease with increasing water content) and the result of microphase separation in the system. By visualizing the distribution of DMSO and water in the volume of the simulation cell, it is possible to extract information about the dependence of the morphology of the water domains on the change in the water fraction in the system. Figure 3B (snapshot 1) shows that PAN and solvent form a homogeneous structure at a low water content (region I in Figure 3A). As the water content increases (*f*_w_ > 0.15), PAN forms a separate phase and water initially forms spherical domains (region II in Figure 3A, snapshot 2 in Figure 3B). Then, with a further increase in water content, the water domains start to coalesce and form elongated dumbbell-shaped structures (region III in Figure 3A, snapshot 3 in Figure 3B). Finally, at a certain threshold (*f*_w_ ≈ 0.8), the domains can coalesce to form a porous structure that percolates throughout the simulated cell (region IV in Figure 3A, snapshot 4 in Figure 3B).

The pores present in the precursor fibers are usually considered defects that promote fiber destruction under high loads in the next drawing stages of PAN production [6,48,49,50]. Thus, the results obtained confirm that one of the possible ways to reduce porosity is to slow coagulation by increasing the amount of good solvent in the system [5,49,50,51,52,53,54,55,56,57,58,59,60].

**Figure 4:**
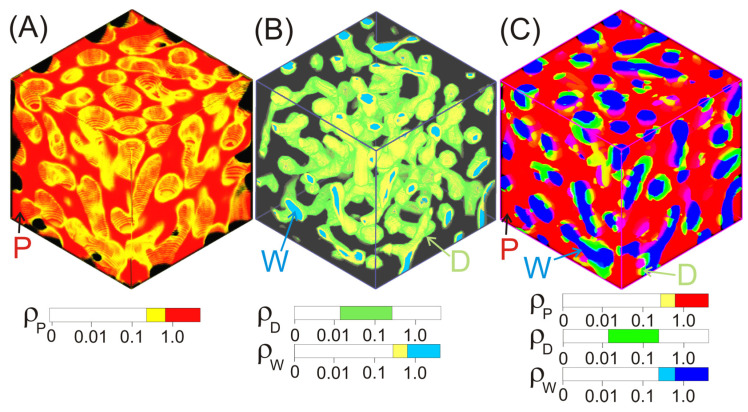
Density distribution within a simulation cell for (**A**) PAN; (**B**) water domains covered by DMSO; (**C**) the combined plot for ρ_P_ > 0.79, ρ_D_ > 0.02, and ρ_W_ > 0.79 (colors correspond to the values determined by the scale bars). *C*_P_ = 80 vol%, *f*_w_ = 0.5, *t*_max_ = 200 Δ*t*.

We have also already discussed above that the DDFT model shows the formation of a “blue cheese” structure that occurs during the coagulation of the spinning solution at a high water content starting from about *f*_w_ ≈ 0.5 (see Figure 1B, Figure 3B and Figure 4C). It is well known that the solvent-filled voids, embedded in the polymer matrix, can form pores during the fiber-drying step. At the same time, regions with the highest PAN density can act as a nucleus for the growth of the crystalline phase in the next stages of the process of drawing and heat treatment of the fiber.

Since the high strength of carbon fibers is limited by internal morphological defects, it is desirable that the precursor PAN fibers also have a homogeneous structure [61]. In order to assess the possible influence of the conditions of the coagulation process on the size of the high-density PAN regions, we have analyzed the final states of the simulated systems at different water contents and fixed volume fraction of PAN (*C*_P_ = 80 vol%). It should be noted that in our DDFT model, we do not deal directly with the packing of polymer chains, but only with the densities of their spatial distribution. By high-density domains, we mean domains where the field density ρ_P_ > 1.03. An example of the visualization of domains with the highest PAN density and their fraction in the volume of the simulation cell is shown in Figures S2 and S3.

**Figure 5:**
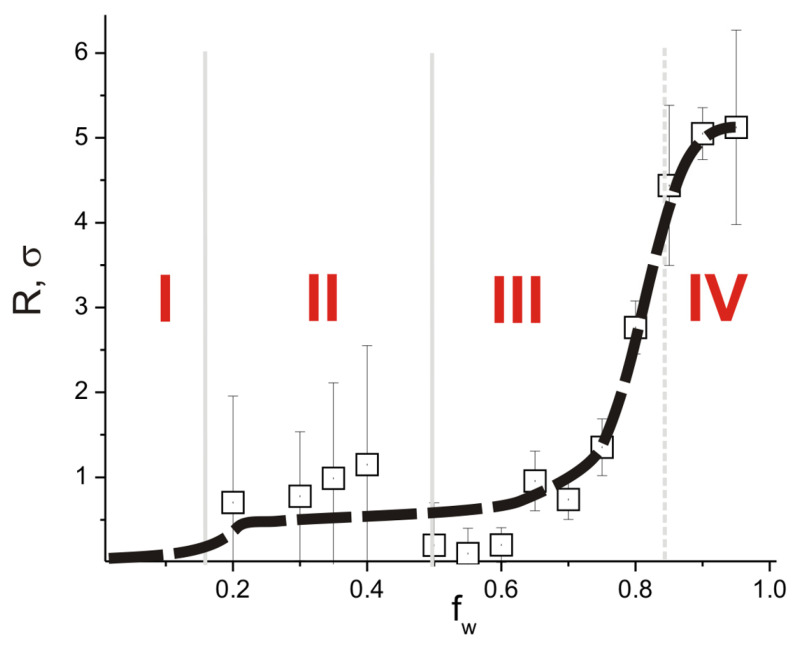
The mean radius of high-density PAN domains (ρ_P_ > 1.03) as a function of the system water content. Roman numbers denote regions where high-density domains (I) do not form, (II) fluctuate strongly in size, (III) increase in size with increasing water in the system, and (IV) stabilize in size. *C*_P_ = 80 vol%.

Figure 5 shows the dependence of the average radius of the high-density domains in PAN on the water content of the system, on which four regions can be identified. At a low water content (region I in Figure 5), there are no dense domains. As the water content increases, PAN begins to form the dense domains, but their characteristics are not stable (region II). This leads to a large error in estimating the size of such regions in samples of the simulated system. With a further increase in the water content, the dense domains stabilize and their average radius increases rapidly (region III). The largest domains of high density are formed in the polymer matrix at the highest water content in the system (region IV). The positions of the regions I–IV in Figure 5 correlate with the positions of the corresponding regions in Figure 3. At the same time, region I (low water content) corresponds to a homogeneous distribution of PAN, DMSO, and water density fields, while region IV corresponds to states where the pores formed by the water form a percolating structure.

If we consider the simulation cell as part of the fiber near-surface layer, the formation of a large number of high-density domains (Figure 5, region IV) can also be interpreted as the development of a rigid shell on the surface of the nascent fibers. Such a shell can significantly slow the mass transfer between the inner regions of the fiber and the coagulation bath. As a result, a core–shell structure is formed (a loose core with capillaries and voids filled with a solvent and a dense surface). The presence of a dense surface layer with numerous nanopores can also cause the fiber cross-section to deviate from a round shape. This can occur as a result of a non-isotropic distribution of nanopores relative to the symmetry axis of the fiber. Thus, our model clearly shows that the formation of precursor fibers with uniform structure is achieved at the high content of the good solvent in the coagulation bath [62,63]. In this way, the behavior of our model agrees with the conclusions of experimental studies and can be used to follow the processes occurring in the fiber both during immersion in the coagulation bath and during the mass transfer process.

## 3. Discussion

Let us discuss how else we can interpret the results of our model. If we arrange the visualizations of the final states of the cells of the system with different PAN contents in the system as a sequence (Figure 1B), a resulting sketch (see Figure S4) of A–C snapshots (I) can be correlated with the change in fiber structure from the surface to the center. At the same time, the set of the same snapshots 1–3 (II), arranged in a different direction in Figure S4, can be identified with the change in a fiber fragment structure over time.

We have already written that the results obtained indicate the formation of a porous structure at the high proportion of non-solvent in the simulation cell (Figure 3B), which is usually considered a defect that weakens the mechanical properties of fibers. On the other hand, high surface porosity may be preferred for applications that require the deposition of a stable color pigment layer on the fiber surface, as well as for the production of filters and sorbents for air/water purification [2,3,4,64]. Additionally, based on the chemistry of the thermal stabilization and the carbonization process [5], it can be assumed that high porosity can be beneficial to better oxygen penetration and the removal of evolved gasses from the fiber during the final stages of CFs production.

It should also be noted that because our model does not work with the specific chemical structures but with the density distributions of their coarse-grained equivalent representations, the results of its predictions are also applicable to other chemical fibers where the Hildebrand solubility parameters of the polymer/combined solvent are close to the values chosen in this study. This can be seen as a disadvantage and an advantage of using the mesoscale simulation technique.

## 4. Materials and Methods

### 4.1. Method and Model

#### 4.1.1. Mesoscale Dynamic Density Functional Theory

We used Dynamic Density Functional Theory to describe the evolution of the PAN/DMSO/water mixture. Details of this method can be found in this list of articles [44,45,46,47,65]. Here, we will only briefly present the main points of this simulation technique.

In DDFT, a model of a molecular system is built in the form of a cubic volume *V*, which is called a simulation cell. Periodic boundary conditions are imposed on the cell faces, allowing the continuum nature of the system to be taken into account. Chemical structures of polymer chains, solvent, and other components of the system are mapped onto a sequence of beads of the same volume *ν*. To do this, we should identify key molecular structures (objects) that play the main role in the behavior of the system. They may be fragments of polymer chains, structural elements of the filler volume, several solvent molecules, etc. As a result, each selected key molecular object in the initial chemical structure is associated with a spherical particle of its own type α in the mesoscale model, α = {1, 2, 3, …}. The value of *ν* is usually evaluated as the average volume of molecular structures corresponding to the beads of the model. Thus, σ = (6*ν*/π)^1/3^ determines the characteristic scale of the model.

Thus, the original molecular structures are replaced by a simplified (coarsened) equivalent representation, which drastically reduces the number of degrees of freedom of the modeled system. The use of coarsening makes it possible to access large-length scales and to run simulations over long time intervals, wherein information about the particular chemical composition of the system is not completely lost. The interaction between the molecular subsystems is controlled by the Flory–Huggins parameters [66,67]. They can be measured experimentally or obtained by classical molecular dynamics (MD) simulations [45].

The distribution of beads of each type is described by time-dependent density fields ρ_α_(**r**,*t*), α = {1, 2, 3*, …*}. Their evolution obeys the Langevin diffusion equations [44,65]

(1) $$\frac{\partial \rho_{\alpha }(r,t)}{\partial t}=D_{\alpha }\nabla^{2}\frac{\delta F[\rho_{\alpha }(r,t)\}}{\delta \rho_{\alpha }(r,t)}+\eta_{\alpha }(r,t),$$

where *F*[ρ_α_(**r**,*t*)] is the free energy functional, *D*_α_ are bead mobility parameters, and η_α_(**r**,*t*) is stochastic noise with a distribution obeying the fluctuation–dissipation theorem [68]. The functional derivative δ*F*[ρ_α_(**r**,*t*)]/δρ_α_(**r**,*t*) is the partial chemical potential µ_α_(**r**,*t*) of the α subsystem at point **r**. Thus, the evolution of the density fields is determined by random “thermal” noise and gradients of chemical potentials arising from the features of the interaction between different molecular subsystems.

In the general case, the system described by Equation (1) is in a non-equilibrium state. Therefore, additional external fields, ω_α_(**r**), are introduced to act on each subsystem. They are chosen so that at each moment of time, *t*, the distribution of densities ρ_α_(**r**,*t*) corresponds to the equilibrium state. For a system consisting of *n*_M_ molecules, the relationship between ρ_α_(**r**,*t*) and ω_α_(**r**) at a fixed time is given by the following partial functional derivative:

(2) $$\rho_{\alpha }[\omega ](r)=-n_{M}k_{B}T\frac{\partial lnZ_{M}[\omega ]}{\partial \omega_{\alpha }(r)},$$

where *k_B_* is the Boltzmann constant, *T* is the absolute temperature, and *Z_M_* is the intramolecular statistical integral. The form of this integral depends on the model representation of the polymer chain. We use the *Z_M_* from [65], which was obtained using the Gaussian model of polymer chains.

The expression for the free energy *F*[ρ,ω] can be written as the following sum:

(3) $$F\left[\rho ,\omega \right]=-k_{B}Tln\frac{\prod_{M}Z_{M}^{n_{M}}\left[\omega \right]}{\prod_{M}n_{M}!}-\sum_{\alpha }\int \omega_{\alpha }\left(r\right)\rho_{\alpha }\left(r\right)dr\;+\;+\frac{1}{2}\sum_{\alpha ,\beta }\iint \varepsilon_{\alpha \beta }(\frac{3}{2}\pi a^{2}{)}^{3/2}exp(-\frac{3}{2a^{2}}\left({r-r^{\prime })}^{2}\right)\rho_{\alpha }\left(r\right)\rho_{\beta }\left(r^{\prime }\right)drdr^{\prime }\;+\;+\frac{1}{2}\kappa \nu^{2}\int {\left(\sum_{\alpha }\rho_{\alpha }\left(r\right)-\sum_{\alpha }{<\rho }_{\alpha }>\right)}^{2}dr,$$

where <ρ_α_> is the average density of the α subsystem, and κ is the compressibility parameter (κ = 10 *k_B_T* [44]). Note that external fields ω_α_(**r**) do not affect intermolecular interactions and are only present in the part of the free energy that relates to the individual molecular subsystems of the model.

The force constants ε_αβ_ in Equation (3) define the intermolecular interactions. They can be calculated from the Flory–Huggins parameter χ_αβ_ using the known relationship [41,67]:

(4) $$\chi_{\alpha \beta }=(\varepsilon_{\alpha \beta }+\varepsilon_{\beta \alpha }-\varepsilon_{\alpha \alpha }-\varepsilon_{\beta \beta })/2\nu k_{B}T,$$

This equation relates the chemical properties of a real system to the interaction energy of coarse particles.

It is should be noted that the problem of calculating chemical potentials is reduced to the problem of calculating external fields ω_α_(**r**) based on the standard self-consistent mean-field method. The implementation of the DDFT method and the corresponding calculation scheme are described in detail in our previous publications [46,47].

#### 4.1.2. Model and Parameterization

To start simulations, we must first: (i) determine the external conditions affecting the system under study, (ii) select the composition of the system, (iii) map all participating molecular objects onto equivalent coarse-grained representations, (iv) determine a characteristic scale of the model, and (v) calculate the parameters of interactions between all molecular subsystems.

Let us describe the principles behind our model construction. When the spinning solution comes into contact with the coagulation bath (the mixture of good solvent and non-solvent in different ratios), the rapid diffusion exchange between the contents of the bath and the spinning solution begins. During this process, the good solvent leaves the nascent fiber while the non-solvent enters it. Phase separation of the combined solvent and polymer then occurs as their miscibility deteriorates. After some time, when the polymer reaches a high density, the mass transfer slows down, and equilibrium conditions are reached between the precipitated polymer and the combined solvent phases. Therefore, the state of the nascent fiber can be considered as a mixture of polymer, solvent, and non-solvent [22]. As a result of the nature of polymers, this is not a true thermodynamic equilibrium, and the final state depends on the conditions of the process used. The specific composition of the system depends on the residence time in the coagulation bath, the chemistry of the spinning solution, the contents of the bath, and its temperature.

Thus, in our model, we assumed that the mass transfer that occurs between the spinning solution and the coagulation bath forms the PAN fiber, and the final state of each fiber fragment modeled is a quasi-equilibrium state. We chose dimethyl sulfoxide and water as good solvents and non-solvents because they are commonly used for the production of PAN fibers [5,12,16,27,28,29,51,69,70,71,72,73,74]. Since the real sizes of nascent fibers of ~60 µm are three orders of magnitude larger than the spatial scale available in DDFT, our studies focus on the processes occurring within a relatively small volume corresponding to different parts of a real fiber. Based on data from the literature [27,29,48,75,76] and the speculations above, we also assume that the nascent fiber consists of a mixture of polymer and residual solvent composition with varying ratios of dimethyl sulfoxide and water.

Since the internal state of our model faciliates multiple interpretations (see Figure S1 (“S” denotes a reference to the Supplementary Materials file)) in our study, we consider a modeling fiber piece to be located near a nascent fiber surface. This means that the simulation cells with different DMSO:water ratios can be understood as states of the system that occur at different compositions of the coagulation bath. This allows us to simulate the effect of different coagulation bath compositions on the structure of the near-surface layer of the fiber by varying the DMSO:water ratio in the cells.

**Figure 6:**
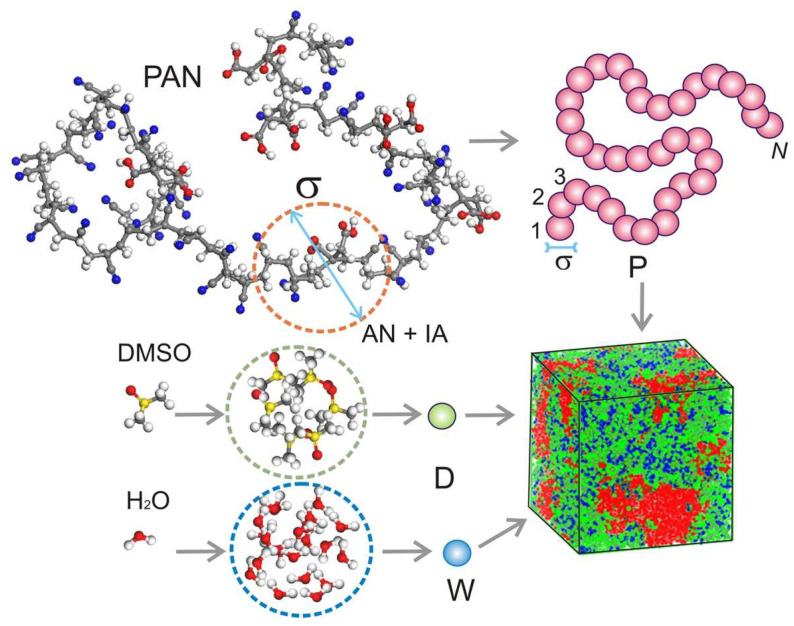
The principle of mapping the atomistic structure of PAN, DMSO, and water to a coarse-grained representation. Beads of type P correspond to the PAN chain segment containing acrylonitrile (AN) and itaconic acid (IA) comonomers, D corresponds to DMSO, and W corresponds to water. The presence of itaconic acid comonomers in the composition of PAN is indirectly taken into account in our model. Colors of atoms in the atomistic model: C—gray; O—red; S—yellow; N—blue; H—white. The following colors of coarse particles (large spheres) and density fields correspond to the color scheme in Figure 1 and Figure 4.

Thus, the modeled system contains three main components: PAN, DMSO, and water. As mentioned above, the DDFT approach assumes that all molecular components of the coarse-grained model consist of spherical particles of the same volume *ν*, and each of the subsystems is assigned its own type of coarse-grained particles. Figure 6 shows the chosen principle of mapping the atomistic structure of the model components to an equivalent coarse-grained representation. P-type beads are used to model PAN chains, D-type beads correspond to DMSO molecules, and W-type beads correspond to water. In this case, a P-type bead is mapped onto a segment of the polymer chain, and D- and W-type beads correspond to multiple DMSO and water molecules, respectively. The choice of volume ν determines the characteristic scale of the system.

In our model, we assume that the spinning solution at the inlet of the coagulation bath has the following composition: PAN—20 vol%; DMSO—80 vol% (volume fractions are used for convenience in setting the composition of the coarse-grained model) [8,16,17,71,72]. We also believe that in the late stages of coagulation, the fiber consists of 80 vol% PAN, 20 vol% DMSO + water [30,72,77,78], because the non-solvent has not completely displaced the good solvent in the freshly coagulated fiber.

#### 4.1.3. Parameters and Characteristic Scale

The intermolecular interaction force constants ε_αβ_ in Equations (3) and (4) are calculated using the Hildebrand solubility parameters δ_α_ [79,80,81], which are related to the Flory–Huggins’ interaction parameters [65,67,79]:

(5) $$\chi_{\alpha \beta }=\frac{\nu {\left(\delta_{\alpha }-\delta_{\beta }\right)}^{2}}{RT}-\chi_{S},$$

where *R* is the gas constant, and χ_S_ is the entropy contribution to the mixing energy, which is usually neglected [67].

The solubility parameters can be obtained experimentally, using atomistic molecular dynamics [45,82] and regression models by Askadskii [83] and Bicerano [83]. Their choice is described in Section S2. Table S1 summarizes the solubility parameters obtained by regression models [83,84], with molecular dynamics (we use a method of Ref. [45], a MULTICOMP package [85] with a polymer consistent force field [86]) and found in literature data [65,78,80,81,82,87,88,89,90,91,92,93,94,95,96]. In our simulations, we use the following parameter values: δ_P_ = 26.4 (J/cm^3^)^1/2^ for PAN, δ_D_ = 26.1 (J/cm^3^)^1/2^ for DMSO, and δ_W_ = 47.8 (J/cm^3^)^1/2^ for water. The reference volume *ν* = 47.8 cm^3^/M is calculated as the average molar volume of PAN, DMSO, and water.

It should be noted that the choice of δ_P_ is based on the fact that although in our model, for simplicity, we consider PAN as a homopolymer, we nevertheless mean that itaconic acid (IA) is present in its composition. The IA comonomers are introduced to PAN to weaken its intermolecular interaction because PAN contains highly polar nitrile groups (-CN), which results in a stiffer chain and an increase in the viscosity of the spinning solution. In addition, the introduction of comonomers affects the cyclization process of PAN. The percentage of comonomers is usually between 2% and 15% [5,15,16]. In our case, we assume that PAN contains 5% of itaconic acid, which determines the correction of δ_P_ relative to the value obtained for PAN homopolymer chains. Thus, using the correction (see Section S2) of the solubility parameters for P-type beads, our model can take into account the multifunctional nature of the polymer chain structure (i.e., the presence of comonomers of several types).

The DDFT model also allows us to take into account the real molecular weights of the polymer chains. A chain length, *N*, of the polymer model is determined by the degree of polymerization and the characteristic ratio *C*_∞_ (the measure of the stiffness of a polymer chain) [79,84]:

(6) $$N=\frac{M_{p}}{M_{mon}C_{\infty }},$$

where *M*_p_ is the molecular weight of the chain, and *M*_mon_ is the molecular weight of the monomer. Using the Bicerano model [84], we obtain for PAN that *C*_∞_ = 7.33. At the same time, the experimental value of *C*_∞_ is in the range of 10.1–18.5 [97]. Thus, assuming *C*_∞_ = 10, for PAN with a molecular weight of 72,000 Da, we have *N* ≈ 136 beads (*M*_mon_ = 53.1 g/M). Since such a model length requires sufficiently large simulation cells, to save computational cost, we reduced the chain length by a factor of 4 to *N* = 34 (the size of an equivalent Gaussian chain is 5.8 σ) and then rescaled all interaction parameters by multiplying them by a factor of 4 [79]. With this assumption, one bead corresponds to ~40 PAN monomers, giving us a characteristic length scale of ~1.82 nm.

At the end of this section, it should be noted that the polymer chain in the DDFT model could also be constructed with explicit consideration of the presence of several types of alternating comonomers. For example, this has been realized in our work [45,46,47], where the size of a monomer was used as the scale unit. In this study, we use large molecular weights of the polymer chains and, at the same time, implicitly take into account the multifunctional structure of the polymer chain, as it has a relatively low percentage of randomly distributed itaconic acid.

### 4.2. Methodology of Simulations

In the course of the simulation, the effect of the ratio of the solvents (DMSO:water) on the structure of the nascent PAN fibers is investigated. All calculations are performed at a fixed temperature of 278 K. The set of Langevin Equation (1) are solved on a regular cubic grid with a total number of nodes *N*_total_ equal to 32 × 32 × 32, with a time step Δ*t* = 0.5τ (τ—time scale). Since the characteristic length scale is σ = 1.82 nm, the step between the nodes for convenience is chosen to be 2 nm. Thus, the selected parameters allow us to study structure formation in a cubic cell with an edge length of 64 nm.

**Figure 7:**
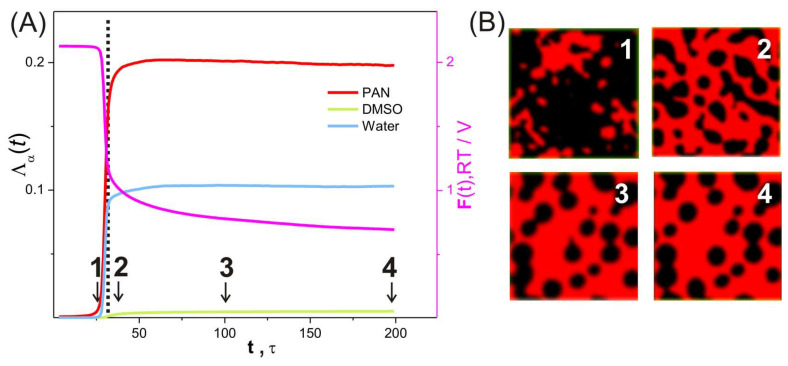
(**A**) Order parameters (red, blue and green lines) and free energy density (pink line) as functions of simulation time. (**B**) Snapshots illustrate the change of the PAN density distribution within the simulation cell in the case of ρ_P_ > 0.8 (red color) at different simulation times. The numbers of the snapshots correspond to the point in time in (**A**). The edge size of the simulation cell is 32σ. The phase separation completion time *t*_ps_ is determined by a linear interpolation of the sharp decrease in the *F*(*t*) function down to the time axis. *C*_P_ = 80 vol%, *f*_w_ = 0.5.

To set up the coefficients *D*_α_ in Equation (1), we used the averaged value of the diffusion coefficients for DMSO and water equal to 3.3 × 10^−11^ m^2^/s [98], which gives the time scale τ of ≈ 121 ns. For all calculations, we use the homogeneous distribution of densities ρ_α_(**r**,0), α = P, D, W as the initial state. The duration of calculations, *t*_max_, is controlled by the free energy convergence condition (|*F*(*t*_2_) − *F*(*t*_1_)| < ε where ε is a small value) (for example, see Figure 7A). The total calculation time, *t*_max_, for each set of parameters, varies from 100 Δ*t* to 10,000 Δ*t*, which is about ≈6 μs to ≈600 μs. It should be noted that the diffusion coefficients values for water and DMSO found in the literature range from 0.22 × 10^−11 ^m^2^/s to 15 × 10^−11^ m^2^/s [27,30,76,98], which allows us to interpret the simulation time range from 1.3 μs to 9091 μs.

Using the chosen values of the solubility parameters, the following values were obtained for the Flory–Huggins interaction parameters: χ_PD_ = χ_DW_ = 0 and χ_PW_ = 9.5. The choice of χ_DW_ should be clarified. If we use the chosen δ_D_ and δ_W_ to calculate χ_DW_, we obtain the value 9.7. This contradicts the known experimental fact about the good miscibility of DMSO and water. Therefore, to eliminate this discrepancy, we chose a zero value for χ_DW_. An estimate of the effect of error in determining the parameters of the interactions shows that small variations in their values have little effect on the radius of the emerging domains.

The volume fraction of PAN *C*_P_ varies in the range from 20 to 80 vol%, and the total volume fraction of the combined solvent DMSO + water *C*_D_ + *C*_W_ is varied from 80 to 20 vol%. The ratio of solvents DMSO:water is controlled by a parameter *f*_w_ = *C*_W_/(*C*_D_ + *C*_W_). Its value is changed from 0 to 1. To avoid the influence of the initial density distribution in the simulation cells, we have performed a series of three calculations using independently generated initial states for each set of parameters. The results obtained are used for averaging.

To control the multiphase separation occurring in the PAN/DMSO/water mixture, we calculate the order parameter Λ_α_. This property is defined as the volume-averaged difference between the square of the local time-dependent density field ρ_α_(**r**,t) and the square of the average density ρ_α_ of particles of type α, respectively:

(7) $$\Lambda_{\alpha }(t)=\frac{\nu }{V}\int {[\rho_{\alpha }^{2}\left(r,t\right)-{<\rho }_{\alpha }>}^{2}]dr.$$

Values of the order parameter close to zero for all subsystems correspond to a system with a uniform distribution of all system components, and Λ_α_ > 0 indicates a phase separation in the system.

Figure 7A shows typical time dependences of the free energy *F* and the order parameters Λ_α_ using the example of the system with *C*_P_ = 80 vol%, *f*_w_ = 0.5. Their shape indicates phase separation induced by non-solvent, the initial phase of which (at *t* < 25 τ) is accompanied by a sharp decrease in the free energy and a rapid increase in the order parameter. To identify the nature of structural changes, we visualize the distribution of specific densities ρ_α_(**r**,*t*) by constructing isosurfaces. An example of such visualization for the PAN-specific density ρ_P_ > 0.8 over time in Figure 7B clearly shows that the system undergoes microphase separation.

**Figure 8:**
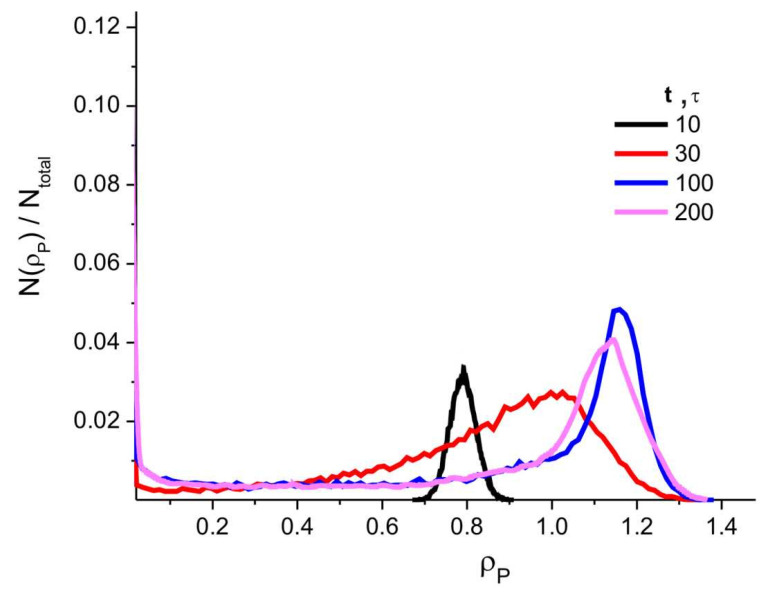
Distribution profiles of the fraction of domains with density ρ_P_ in the simulation cell at different simulation times (*N*(ρ_P_) is the number of nodes with a density equal to ρ_P_ = 0–1.4, and *N*_total_ is the total number of nodes in the grid of the simulation cell). *C*_P_ = 80 vol%, *f*_w_ = 0.5.

The intense structural changes occur at characteristic times close to the time *t*_ps_ of phase separation (coagulation) (Figure 7B, snapshots 1, 2, and 3). The time of phase separation *t*_ps_ is estimated by linear extrapolation of the region of rapid change in the free energy of the system along the time axis (see Figure 7A). After *t* > 50 τ, the values of Λ_α_ (α = P, D, W) slowly tend towards saturation (Figure 7A), the structure is still evolving, but the changes that occur are (essentially) small (Figure 7B, snapshots 3 and 4). This can also be seen in Figure 8, which shows the values of a certain fraction of PAN regions with different densities of ρ_P_, *N*(ρ_α_)/*N*_total_, where *N*(ρ) is the number of nodes with a density equal to ρ, and *N*_total_ is the total number of grid nodes in the simulation cell, at different simulation times. Therefore, we stop our simulation after the end of the microphase separation (*t*_max_), when the values of *F* and Λ_α_ reach saturation (Figure 7A). The time of this event depends on the composition of the system and varies in our calculations from 100 to 10,000 integration steps of Equation (1).

At the final state, *t* >> *t*_PS_, a dense porous structure of water (the “blue cheese” type) with an average pore cross-section size of ≈12 nm is present in the PAN matrix (Figure 7B). The pores are filled with a mixture of DMSO and water. This is confirmed by the combined plot of the maximum values of the densities ρ_α_ (α = P, D, W), shown in Figure 4. It can be seen that PAN and water form an unoriented fibrillar 3D network structure with regions of high-density PAN matrix and water domains.

## 5. Conclusions

The article presents a coarse-grained model based on the method of dynamic density functional theory. It aims to study the structure formation caused by the occurrence of phase transitions in solutions based on polyacrylonitrile. This model allows us to observe the processes of phase separation in the nascent fiber as a result of the coagulation of the spinning solution in the coagulation bath. The calculations performed allow us to follow the changes in the internal structure of the system and estimate the size of the structural features.

In general, the model adequately describes the phase separation processes occurring in the system as a function of the solvent quality, which is controlled by the water content in the combined solvent. Several conclusions can be drawn from the results obtained. By changing the composition of the coagulation bath, it is possible to control the fiber morphology. The greatest inhomogeneity of the PAN fiber structure occurs at a high water content in the combined solvent. In this case, a large number of high-density domains are also formed. Conversely, under conditions where a good solvent is present, a homogeneous spatial structure of the polymer matrix is formed. The latter can be favorable for multi-stage fiber drawing, helping to achieve high orientation with a high degree of crystallinity.

The model developed in this work can be used to optimize the structure of PAN nascent fibers and to better understand the kinetics of the processes involved in the formation of chemical fiber structures based on other polymers. Although our model has limitations, taking into account the complexity of the phenomena occurring during the formation of the structure of the nascent fiber (from the point of view of the theoretical description), it nevertheless gives an adequate physical picture with the assumptions made. Therefore, the results obtained give reason to expect that we have obtained an additional method to analyze the formation of chemical fibers.

## Data Availability

Data sharing is not applicable.

## Supplementary Materials

The following supporting information can be downloaded at: https://www.mdpi.com/article/10.3390/ijms24119312/s1.
