# Supplementary material for: Mesoscale Simulations of Structure Formation in Polyacrylonitrile Nascent Fibers Induced by Binary Solvent Mixture
Source: Int J Mol Sci. 2023 May 26;24(11):9312. doi: 10.3390/ijms24119312 (PMC10253059; doi:10.3390/ijms24119312)
Supplement: Supplementary file 1 [file ijms-24-09312-s001.zip › ijms-2416354-supplementary.pdf]

Mesoscale Simulations of Structure Formation in Polyacrylonitrile Nascent Fibers Induced by Binary Solvent Mixture

P.V. Komarov[1,*], M.D. Malyshev[1], P.O. Baburkin[1], D.V. Guseva[2]

[1] Scientific Research Department, Tver State University, Zhelyabova 33,170100 Tver, Russia;

[2] A.N. Nesmeyanov Institute of Organoelement Compounds RAS, Vavilova St. 28, 119991 Moscow, Russia

Corresponding author:
*E-mail: komarov.pv@tversu.ru (P.V. Komarov)

Here we present an additional set of data from a computer simulation of the system under investigation.

S1. Model

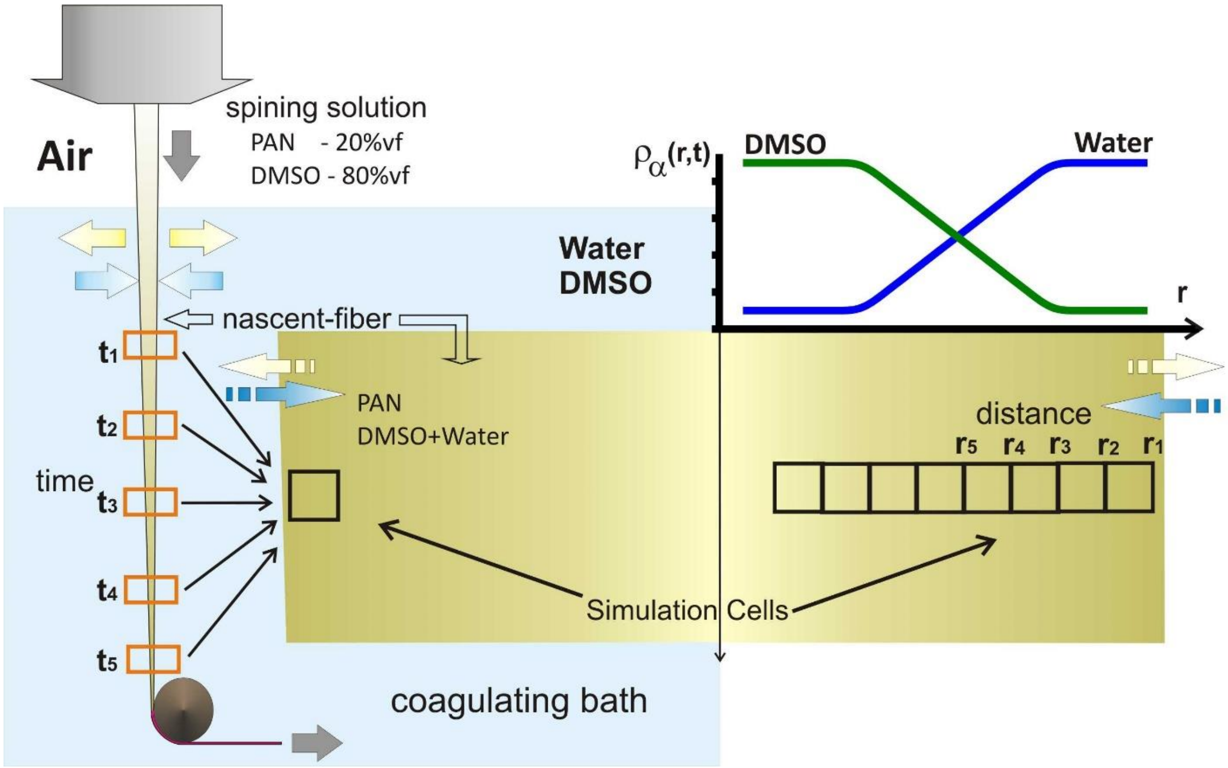

Figure S1. Sketch of the dry-jet wet spinning process and interpretation of the internal states in the simulation cell. It could be assumed that fragments of a nascent fiber are located at different distances from the surface (the farther the simulation cell is from the surface, the greater the volume fraction of dimethyl sulfoxide (DMSO) it contains). Alternatively, it could be assumed that the simulation cell is located close to the fiber surface. Thus, the change in the DMSO:water ratio can be considered as 1) a quasi-stationary state of the system at different stages of the coagulation process, or 2) the effect of the coagulation bath composition on the fiber structure at a fixed distance from the center.

S2. Solubility Parameters

To parameterize the interaction between the subsystems of the model (Equation (4) and (5)), we chose the values of the Hildebrandt solubility parameters [80,81] and the molecular volumes for polyacrylonitrile (PAN), itaconic acid (IA), dimethylsulfoxide (DMSO) and water based on theoretical estimates and data found in the literature. Note that all references are listed in the main text of the article.

The semi-empirical regression models proposed by Askadskii [83] and Bicerano [84] can be used to quickly estimate solubility parameters. For more accurate results, calculations within the framework of the atomistic molecular dynamics (MD) method [45-47] can be performed using the cohesive energy density E_{coh}/V according to the ratio

$$\delta_{PAN} = (E_{coh}/V)^{1/2}, \tag{S1}$$

V – a simulation cell volume. In MD, the E_{coh} value characterizes the intensity of intermolecular interactions and can be interpreted as a change in the potential energy of the system as molecules pass from the condensed state to the gas phase. In practice, this requires extensive scanning of a large volume of configuration space to obtain a representative sample of molecular configurations characteristic of the equilibrium structure of the material.

To calculate E_{coh}, we applied the modeling technique of Ref. [45,82], implemented within the MULTICOMP package [85] with the second-generation Polymer Consistent Force Field (PCFF) [86]. Because the cohesion energy is sensitive to the accuracy of determining the density of the simulated substance, we used experimental densities to construct samples of all systems. For each molecular system, samples with different numbers of molecules n = 4, 16, 32, and 64 were constructed to improve the accuracy of the calculations. The cohesive energy density was evaluated by extrapolating $E_{coh}(n)/V(n)$ to the limit when $n \to \infty$. To exclude the influence of initial-state peculiarities, three statistically independent initial states were constructed for each system. The solubility parameters for PAN (δ_{PAN}) and IA (δ_{IA}) were calculated using short oligomeric chains composed of four acrylonitrile monomers. The values obtained are summarized in Table S1.

As can be seen in Table S1, in comparison with the experimental results, Askadsky's regression model gives a slightly underestimated value for the solubility parameter for PAN and water, reproduces the value for DMSO, and overestimates for itaconic acid. In the case of molecular dynamics, compared with the experiment, it underestimates the values for PAN and DMSO and reproduces well the values for itaconic acid and water. In general, both theoretical approaches reproduce the general trend $\delta_{PAN} \approx \delta_{IA} < \delta_{H2O}$, which makes it possible to use these approaches to determine the solubility parameters in the absence of experimental data.

Since there are many experimental data for PAN, IA, DMSO, and water, we chose the following values averaging them for the parameterization of the model: δ_{PAN} = 26.6 [J/cm^3]$^{1/2}$, δ_{IA} = 22.6 [J/cm^3]$^{1/2}$, δ_{DMSO} = 26.1 [J/cm^3]$^{1/2}$ and δ_{water} = 47.8 [J/cm^3]$^{1/2}$. The proximity of δ_{PAN} and δ_{DMSO} values means that DMSO is a good solvent for PAN, which is a well-known fact [78,89]. At the same time, the large difference between the values of water and DMSO parameters ($\delta_{DMS} << \delta_{H2O}$)

contradicts the fact of miscibility of DMSO with water. They mix due to the strong interaction of water with the >S=O group of DMSO. Therefore, when parameterizing the interaction of water and DMSO, one should introduce a correction of the parameters of the interaction of water and DMSO.

Table S1.

Density, molecular volume (V_m), molar mass (M_m), and the Hildebrant solubility parameters (δ).

	Density [g/cм^3]	δ [J/cm^3]$^{1/2}$	V_m [cm^3/M]	M_m [g/M]	δ [J/cm^3]$^{1/2}$	Density [g/cм^3]	δ [J/cm^3]$^{1/2}$
**Chemical compound**	**Ascadskii**				**MD**	**Literature data**	
PAN*	1.11	24.4	47.7	53.1	18.7±0.1	1.14 [78]	27.4 [78], 27.4 [80], 25.5 [81], 27.43 [88], 25.3 [96]
IA**	1.14	31.5	91.3	130	23.8±0.6	1.63 [87]	20.97, 24.23 [93]
DMSO	1.03	27.4	75.8	78.1	18.4±0.3	1.1 [80,82]	26.7 [78], 26.67 [80], 29 [82], 22.14 [89], 26.07 [90], 26.4 [92], 24.6, 26.4 [94], 26.6 [96]
Water	1	40.9	17.7	18	47.1±0.1	1	47.9 [65], 47.8 [80], 47.50 [91], 48.0 [92], 47.9 [94,95]

With the help of the Bicerano model, we can obtain alternative estimates:
*) for PAN δ_{PAN} = 24.5 [MPa]$^{1/2}$.
**) for IA δ_{IA} = 26.9 [MPa]$^{1/2}$.

In addition to the correction for the DMSO-water interaction parameters, a correction for the PAN solubility parameter should be introduced. The following explanation can be made. In our model, we have assumed that PAN contains itaconic acid as a comonomer with a random distribution along the chain. Therefore, a correction for δ_{PAN} has to be made and can be done using a simple rule of additivity [95]

$$\delta_{PAN+IA} = (1-\phi)\, \delta_{PAN} + \phi\, \delta_{IA}\ , \qquad (S2)$$

here ϕ is the volume fraction of itaconic acid. For a more accurate correction (as an alternative), you can also use the following expression [95]

$$\delta_{AN+IA}{}^2 = (1-\phi)\, \delta_{PAN}{}^2 + \phi\, \delta_{IA}{}^2 - \Delta H_{mix}/((1-\phi)\, V_{PAN} + \phi\, V_{IA}), \qquad (S3)$$

here ΔH_{mix} is the value of the mixing enthalpy of the components of the binary system and V_α is the molar volume. According to the data from the literature, the proportion of itaconic acid monomers in the composition of PAN can vary from 2% to 15%. Thus, using (S2), we can see that δ_{PAN+IA} can vary in the range of 26 to 26.5 [J/cm^3]$^{1/2}$. To parameterize the model, we used δ_{PAN+IA} = 26.4 [J/cm^3]$^{1/2}$, which corresponds to 5% itaconic acid. Thus, by correcting for the solubility parameters in our model, we can take into account the multifunctional nature of the polymer chain structure.

S3. Extra results

A radius of PAN areas with high density is evaluated as $R = (3V/4\pi)^{1/3}$. Volumes, V, of such areas were calculated by counting the number of nodes in the simulation cell where $\rho > 1.3$.

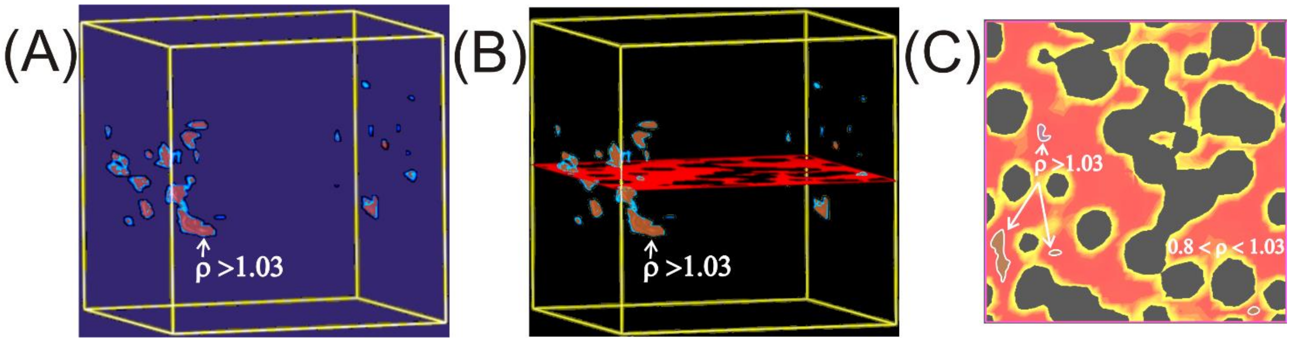

Figure S2. Instances of visualization areas with the highest density in the PAN matrix when $\rho > 1.03$; A) within the entire simulation cell; B) the simulation cell with a cross-section at (x,y,0); C) a top view of the cross-section shown in Figure "B"; C_P = 80 vol%, f_w=0.5.

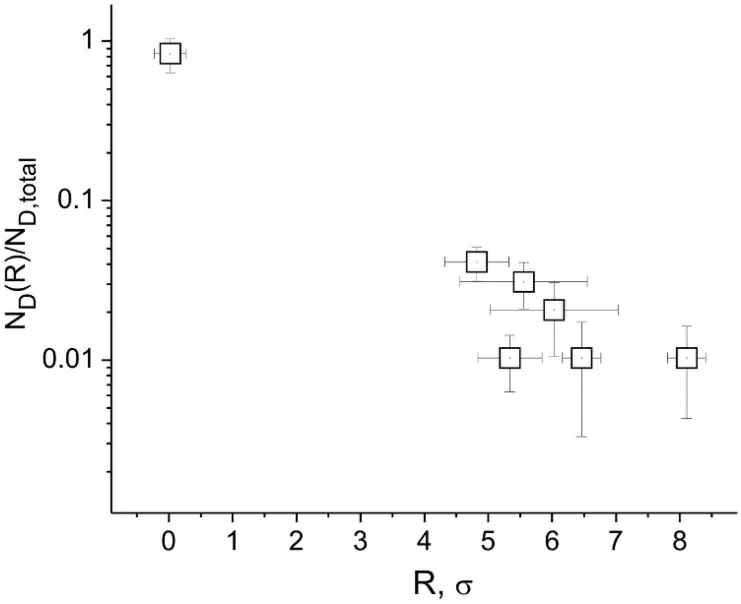

Figure S3. The instance of the distribution of the fraction of the PAN matrix regions with radius *R*. C_P = 80 vol%, f_w= 0.9.

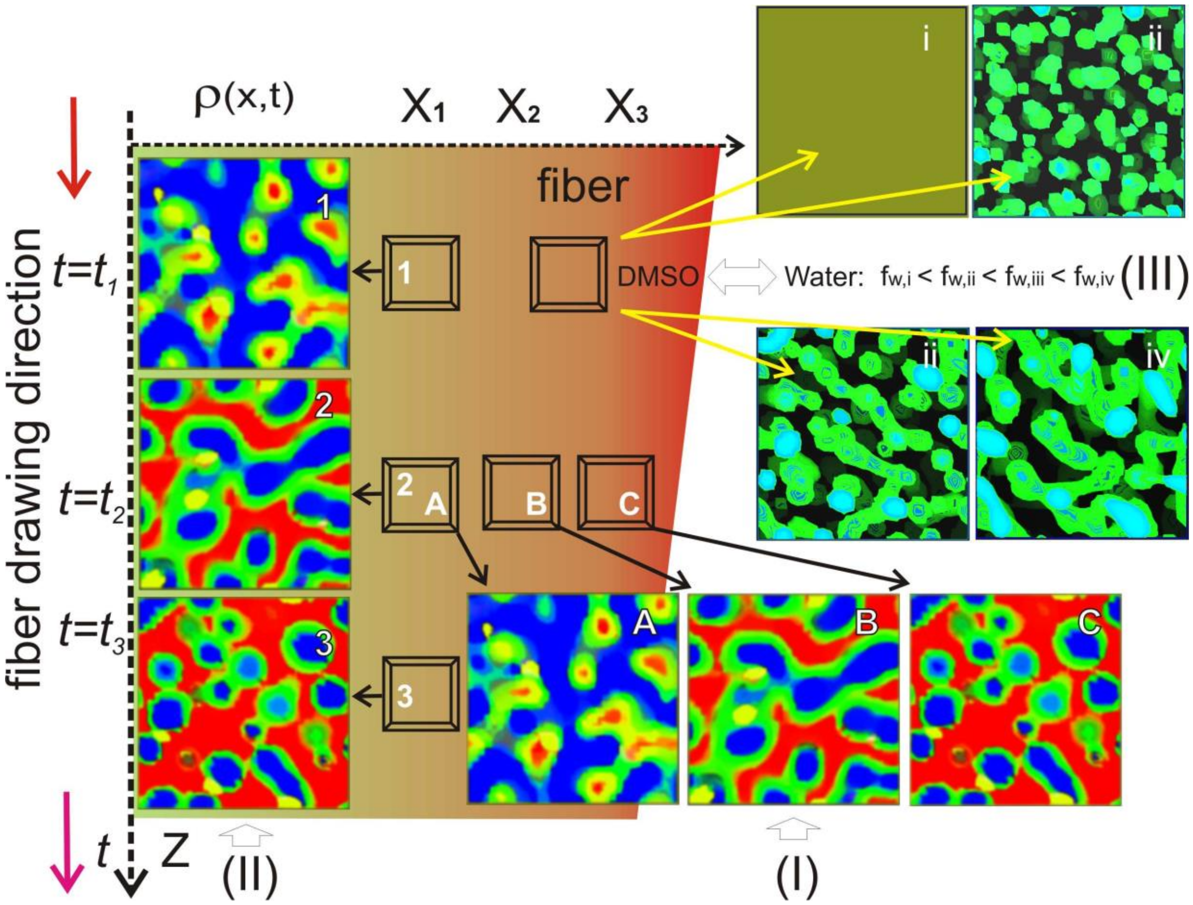

Figure S4. Visualization of the possible interpretation of the fiber model. Simulation cells in ascending order: I) fragments of a nascent fiber at different distances from the surface; II) at different times as the fiber moves in the coagulation bath; III) fragment of a nascent fiber at a fixed distance from the surface (this sequence describes the effect of the composition of the coagulation bath on the fiber structure at a fixed distance from the center).
